# Is the Pinball Machine a Blind Spot in Serious Games Research?

**DOI:** 10.2196/72354

**Published:** 2025-04-02

**Authors:** Jens Peter Eckardt

**Affiliations:** 1 Research Unit/Knowledge Center (Videnscenter), Bedre Psykiatri, Gammeltorv 14. 2 sal, Copenhagen, 1457, Denmark, 45 28943288

**Keywords:** serious games, research, interventions, arcade technology, digital game paradigm, pinball gaming, arcade gaming, executive functions, neurodiversity, cognitive training, therapeutic interventions

 In their insightful and comprehensive study, Rodríguez Timaná et al [1] illuminate the impact and effectiveness of serious games on executive functions and their integration into educational and therapeutic settings for neurodiverse populations aged 3‐22 years, using various technologies. However, by focusing on conventional and emerging technologies like virtual reality, smartphones, tablets, PCs, and cameras or sensors, one may overlook long-established technology such as the pinball machine. Its enduring presence in the gaming industry, with a rich history of gameplay designs, mechanics, electronics, and both kinetic and digital formats, raises questions. Could pinball’s format, rooted in both physical and digital realms, also bridge the gap between traditional and modern approaches to serious gaming? Could it offer a more tangible, interactive experience as a promising therapeutic tool (or as an adjunct) compared to conventional serious games?

For decades, numerous studies of varying quality and methodologies have examined the use of pinball machines as an intervention for individuals with conditions such as autism, severe disabilities, intellectual disabilities, visual impairments, and Parkinson disease. In 1961, the actions of two autistic children were recorded in an experiment involving devices like a pinball machine, aiming to demonstrate how reinforcement worked and to control their behavior [2]. In 1981, a study concluded that adolescents with severe and profound intellectual disabilities could effectively learn and generalize the use of an electronic pinball machine as a leisure skill [3]. In 2021, Luszeck [4] demonstrated the positive benefits of pinball for individuals with Parkinson disease, showing improvements in balance, fine motor skills, and quality of life. In 2022, Johnson et al [5] explored the mental health benefits of pinball for men (including participants with attention-deficit/hyperactivity disorder), revealing improved well-being both directly and through social connections.

Pinball is a game that engages multiple cognitive processes, strengthening executive functions. Players must maintain attention on the ball and targets. The ball moves quickly, requiring focus to react in real time, thus enhancing sustained attention. Pinball involves quick decision-making under pressure. Players control impulses and emotions, making strategic decisions about when and how to launch the ball or use the flippers, thus improving self-regulation. Players also need to remember the board layout, previous actions, and strategies to maximize points, tapping into working memory and planning skills. Pinball requires players to adjust strategies in response to unpredictable events (eg, the ball bouncing off targets), exercising cognitive flexibility. Players anticipate ball movement and plan for upcoming moves, supporting problem-solving skills. Frequent switching of focus between parts of the machine (flippers, bumpers, targets, and video) helps develop task-switching abilities.

Why is pinball gaming and technology underexplored in serious game research, including theoretical exploration, despite its ongoing development? Is it less adaptable or perhaps too costly, compared to other technologies? While pinball is considered entertainment, the cognitive demands it places on players may provide an effective way to exercise executive function skills. To fully harness its potential as a serious game, researchers must expand their focus, paving the way for arcade applications such as the pinball machine within the digital-centric paradigm.
